# Adherence to the Paleolithic diet and Paleolithic-like lifestyle reduce the risk of colorectal cancer in the United States: a prospective cohort study

**DOI:** 10.1186/s12967-023-04352-8

**Published:** 2023-07-19

**Authors:** Yi Xiao, Yaxu Wang, Haitao Gu, Zhiquan Xu, Yunhao Tang, Hongmei He, Linglong Peng, Ling Xiang

**Affiliations:** 1 Department of Gastrointestinal Surgery, The Second Affiliated Hospital of Chongqing Medical University, No.288, Tianwen Avenue, Nan’an District, Chongqing, 400010 China; 2 Department of Clinical Nutrition, The Second Affiliated Hospital of Chongqing Medical University, Chongqing, China

**Keywords:** Paleolithic diet, Paleolithic-like lifestyle, Colorectal cancer, Epidemiology, Cohort study

## Abstract

**Background:**

The plant-based paleolithic diet (PD) and the paleolithic-like lifestyle (PLL) may reduce the risk of chronic diseases, including colorectal adenomas. These dietary and lifestyle approaches are proposed to exert their effects through mechanisms such as reducing inflammation, oxidative stress, and insulin levels. However, whether PD and PLL is associated with the risk of colorectal cancer (CRC) has not been determined.

**Methods:**

A cohort of 74,721 individuals who participated in the PLCO study were included in this analysis. Adherence to the PD and PLL was assessed using PD and PLL scores, where higher scores indicated greater adherence. Multivariable Cox models were utilized to estimate hazard ratios (HRs) and 95% confidence intervals (CIs) for the risk of CRC and its subsites (proximal colon cancer and distal CRC). Subgroup analyses were conducted to identify potential effect modifiers.

**Results:**

During a mean follow-up of 9.2 years, a total of 694 CRC cases were identified. Participants in the highest compared with the lowest quartiles of PD score had a lower risk of CRC (Q4 vs Q1: HR 0.76, 95% CI 0.61–0.95, *P*_*trend*_ = 0.009) and proximal colon cancer (Q4 vs Q1: HR 0.73, 95% CI 0.55–0.97, *P*_*trend*_ = 0.02). A stronger inverse association was observed for PLL score with the risk of CRC (Q4 vs Q1: HR 0.64, 95% CI 0.51–0.81, *P*_*trend*_ < 0.001), proximal colon (Q4 vs Q1: HR 0.62, 95% CI 0.46–0.83, *P*_*trend*_ = 0.001) and distal CRC (Q4 vs Q1: HR 0.69, 95% CI 0.48–0.98, *P*_*trend*_ = 0.03). Subgroup analyses revealed the inverse association of PD score with the risk of CRC was more pronounced in participants with BMI < 30 (Q4 vs Q1: HR 0.68, 95% CI 0.53–0.87) than in those with BMI ≥ 30 (Q4 vs Q1: HR 1.07, 95% CI 0.68–1.67) (*P*_interaction_ = 0.02).

**Conclusions:**

Our findings suggest that adhering to the PD and PLL could be a new option to reduce CRC risk.

**Supplementary Information:**

The online version contains supplementary material available at 10.1186/s12967-023-04352-8.

## Introduction

Colorectal cancer (CRC) is a significant global public health concern, ranking as the third most common cancer in men and the second most common cancer in women [1]. In the United States alone, CRC is estimated to cause 150,000 new cases and 50,000 deaths in 2023 [2]. The etiology of CRC is multifactorial, and research has established that over 50% of cases and deaths can be attributed to modifiable risk factors, including unhealthy dietary habits, smoking, heavy alcohol consumption, physical inactivity, and obesity [3]. Therefore, adopting a healthy diet and lifestyle may play a crucial role in reducing the incidence of colorectal cancer.

In recent years, there has been growing interest in the Paleolithic Diet (PD) and Paleolithic-like lifestyle (PLL) within the field of nutritional epidemiology [4]. Previous study has suggested that evolutionary inconsistencies, stemming from differences in diet and lifestyle between Paleolithic Homo sapiens and modern Homo sapiens, may be a contributing factor to the significant rise in chronic diseases observed over the past century [4]. In brief, the PD is a dietary pattern that is estimated based on anthropological evidence from fossils and existing hunter-gatherer groups, and is typically characterized by a focus on plant-based foods, such as a variety of fruits and vegetables, along with wild plant foods that are high in calcium and other minerals [5]. The diet is also typically rich in nuts and lean meats, while avoiding or minimizing dairy products, grains, sugar, and salt. Whalen et al. developed the PD score based on the specific characteristics of this diet [6, 7], while Sohouli et al. expanded upon this score by incorporating lifestyles factors that have been shown to have a significant impact on disease [8]. By integrating physical activity level, body mass index (BMI), and smoking into the PLL score, Sohouli et al. provide a more comprehensive assessment of adherence to the Paleolithic lifestyle. Together, these two scores can help researchers and clinicians evaluate the impact of the Paleolithic diet and lifestyle on health outcomes. In fact, many studies have used the PD score alone or in combination with lifestyle factors (i.e., PLL score) to analyze the incidence of chronic diseases [8, 9], cancer [10, 11], and mortality [7]. In particular, Whalen et al. found that higher adherence to PD was associated with a lower incidence of colorectal adenomas [6]. Given that colorectal adenomas are often considered to be precursors of CRC [12], there may be a potential association between PD and CRC risk.

To our knowledge, no observational studies have examined the potential association between PD and PLL and the risk of CRC. Hence, to fill this gap, the present study comprehensively analyzed the potential association of PD and PLL with the risk of CRC and its different anatomical subsites in a large population-based cohort.

## Methods

### Study design

Our study population was derived from the Prostate, Lung, Colorectal, and Ovarian (PLCO) Cancer Screening Trial, a randomized controlled study of screening exams or tests for PLCO cancers. The aim of this trial was to test whether these screening exams or tests could lower the risk of death from these cancers. Study design of the PLCO Cancer Screening Trial has been reported previously [13]. Briefly, between 1993 and 2001, a total of 154,887 men and women between 55 and 74 years of age were finally enrolled in this trial from 10 screening centers across the US [14]. Enrolled individuals were individually and randomly divided into the control group or the arm group in equal, with those received either standard care or CRC screening (sigmoidoscopy, insertion to at least 50 cm with 90% of mucosa visible or a suspect lesion identified) separately [15]. All participants in the PLCO Cancer Screening Trial provided written informed consent, and the study was approved by the US National Cancer Institute and the Institutional Review Board of each screening center.

**Fig. 1:**
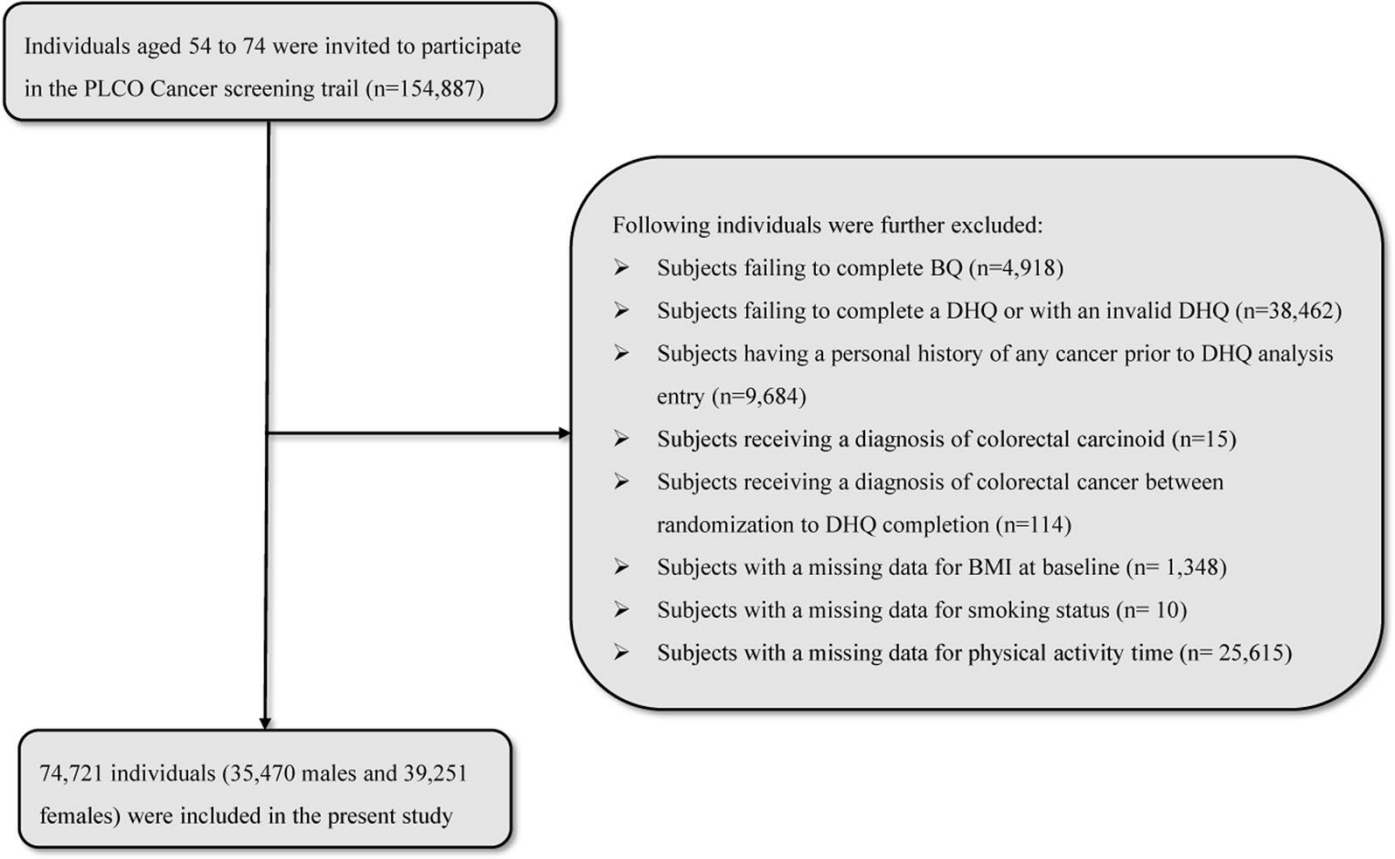
The flow chart of identifying eligible subjects. *PLCO* Prostate, Lung, Colorectal, and Ovarian, *BQ* baseline questionnaire, *DHQ* diet history questionnaire

In the present study, the following individuals were further excluded: (1) Failure to complete the baseline questionnaire (BQ) (n = 4918); (2) Returned a invalid dietary history questionnaire (DHQ), which was defined as lacking a completion date, completed DHQ after the death date, with a high frequency of missing responses (≥ 8), or have extreme values of calorie intake (defined as the first or last percentile) (n = 38,462); (3) Individuals with any type cancer history before the DHQ analysis entry (n = 9684); (4) Individuals with a diagnosis of colorectal carcinoid (n = 15); (5) A total of 114 individuals with any outcome event during the period between randomization and completion of the DHQ (outcome events included the development of colorectal cancer, death, or lack of a follow-up analysis); 6) Individual with a missing data of BMI at baseline (n = 1348), smoking status (n = 10), and physical activity time (n = 25,618), which type data should be used to construct the PLL score. Ultimately, a total of 74,721 individuals, including 35,470 males and 39,251 females, were included in this study, as presented in Fig. 1. The PLCO Screening Trial project obtained approval from the Institutional Review Board of the National Cancer Institute (NCI) as well as from each of the participating screening centers (https://biometry.nci.nih.gov/cdas/plco/). All individuals involved in the trial provided explicit, informed, and written consent. Additionally, our study (Project ID: PLCO-1215) has received approval from the the United States NCI.

### Data collection

In this study, baseline data were collected using a self-administered baseline questionnaire (that is, BQ), which included age, sex, race, education level, smoking status, BMI at baseline, diabetes, diverticulitis or diverticulosis, colon comorbidity, colorectal polyp, aspirin use, and a family history of colorectal cancer. Notably, BMI was calculated as weight in kilograms divided by the square of height in meters. Dietary intake information, including alcohol consumption, dietary energy intake, and dietary foods or nutrients intake, were collected using a 137-item self-administered food frequency questionnaire (FFQ) called the DHQ over a 3-year period after enrollment. Daily food intake was estimated by calculating the product of food frequency and portion size. Estimates of daily energy and nutrient intake were based on the United States Department of Agriculture's 1994–1996 Continuing Survey of Food Intakes by Individuals [16], which was commonly used nutrient database. In addition to this, DHQ recorded the age of individuals when they completed the DHQ and calculated the Healthy Eating Index-2015 (HEI-2015) as described in the literature [17], which is used to assess the dietary quality of individuals. Previous research, such as the Eating at America's Table Study, had substantiated the validity of the DHQ in dietary assessment [18]. Physical activity was assessed using a self-reported supplemental questionnaire (SQX), which evaluated the total weekly time spent engaging in moderate to high-intensity exercise.

### Paleolithic diet and paleolithic-like lifestyle score calculation

In this investigation, the PD score and PLL score were formulated according to the method utilized by Sohouli et al. [8]. In brief, all food items identified in the DHQ were categorized into 14 predetermined food groups based on their nutrient and culinary similarities. These food groups were subsequently classified into two overarching categories, namely those with PD characteristics (comprising of vegetables, fruits, a score for fruit and vegetable diversity, lean meat, fish, nuts, and calcium) and those without PD characteristics (including red and processed meat, dairy products, sugar-sweetened beverages, baked goods, grains and starches, sodium, and alcohol), which as presented in Table 1. The score of fruit and vegetable diversity was defined as the number of components of the fruit and vegetable group consumed by each individual. Furthermore, to consider dietary calcium separately from dairy products, we used the residuals of a linear regression of total calcium intake on total dairy food intake to represent calcium intake independent of dairy consumption since the Paleolithic diet had little dairy food but high amounts of calcium (from wild greens) [5]. Participants were grouped into five strata based on quintiles of consumption of individual food groups and were awarded a score ranging from 1 to 5 points according to the following criteria: (1) those food groups with PD characteristics were positively scored (with “1” indicating the minimum consumption in each food group and “5” indicating the maximum consumption); (2) while those food groups without PD characteristics were inversely scored. Ultimately, the PD score for each participant was calculated as the sum of points awarded for each food group, with a possible range of 14 to 70 points, where higher scores indicated a greater adherence to the PD.

**Table 1:** Constituents and Construction of the Paleolithic Diet (PD) score ^a^

Dietary food components	Paleolithic diet score^b^	Dietary foods intake^c^
More characteristic of diet		
Vegetables	Highest intake “best”	280 ± 180
Fruit	Highest intake “best”	180 ± 150
Fruit and vegetable diversity score	Highest intake “best”	26 ± 3
Lean meat ^d^	Highest intake “best”	66 ± 47
Fish	Highest intake “best”	16 ± 18
Nuts	Highest intake “best”	7 ± 15
Calcium	Highest intake “best”	1020 ± 520
Less characteristic of diet		
Red and processed meat^e^	Lowest intake “best”	12 ± 15
Dairy foods	Lowest intake “best”	270 ± 280
Sugar sweetened beverages	Lowest intake “best”	390 ± 460
Baked goods^f^	Lowest intake “best”	26 ± 28
Grains and starches^g^	Lowest intake “best”	120 ± 89
Sodium	Lowest intake “best”	2700 ± 1200
Alcohol	Lowest intake “best”	10 ± 25

a All components of food were in grams/day except calcium and sodium which were in milligrams/day. Highest intake “best”: points were assigned to each quintile; highest and lowest quintiles scored 5 and 1 points, respectively. For lowest intake “best”, the scoring was reversed; highest and lowest quintiles scored 1 and 5 points, respectively

b The PD score had 14 components and possible scores ranged from 14 to 70

c Values are mean ± standard deviation (grams/day)

d Lean meats included skinless chicken or turkey and lean beef

e Red and processed meat included sheep, pork, beef, ham, bacon, sausage, hot dogs and so on

f Baked goods included items such as cake, pie, and other pastry-type foods

g Grains and starches included different sources of grains and starches including potatoes

According to the method used by Sohouli et al. [8], the PLL score is a combination of physical activity score, BMI score, smoking status score and PD score. In this study, a score of 5 was assigned to individuals in the highest tertile of physical activity, while scores of 3 and 1 were assigned to those in the middle and lower tertiles, respectively. The scoring scheme was reversed for BMI. Smoking status was scored as 5, 3, and 1 for non-smokers, ex-smokers, and smokers, respectively. The scores of the PD and the above three lifestyle factors were then combined to compute the PLL score for each individual. The final score range in our study was from 17 to 85, with higher scores indicating greater adherence to the PLL.

### Ascertainment of CRC

In this study, the identification of CRC cases relied mainly on an annual study update form. The screening centers mailed the annual form to each living participant, requesting information about any cancer diagnosis they received, including the site, type, date, location of diagnosis, and contact information for their healthcare providers. To ensure the accuracy of the reported cancer cases, relevant medical records were reviewed using a standardized form. Study physicians, who were blinded to participants' risk factors, confirmed the cases and their anatomical locations. In study, CRC were defined based on the definitions by the International Classification of Diseases for Oncology (ICD-O; codes: colon cancer: C18, and rectal cancer: C19-C20). Proximal colon cancers including cecum, appendix, ascending colon, hepatic flexure, transverse colon, and splenic flexure colon cancer. Distal CRC including descending cancer, sigmoid colon cancer, rectosigmoid junction cancer and rectal cancer. It is worth noting that the primary outcome in this study was CRC, and the secondary outcome was proximal colon cancer and distal CRC.

### Statistical analysis

To minimize potential biases and increase statistical power, the following strategies were employed to impute missing data for seven variables with missing proportions of less than 5%. We utilized the mode and median methods to impute categorical and continuous variables in the presented study, respectively. The imputed variables comprised education level, diabetes, aspirin use, diverticulitis or diverticulosis, colon comorbidity (including ulcerative colitis, Crohn’s disease, Gardner's syndrome, and familial polyposis), colorectal polyp, and family history of colorectal cancer. Additional information on the specific types and proportions of missing data can be found in Additional file 1: Table S1.

**Fig. 2:**
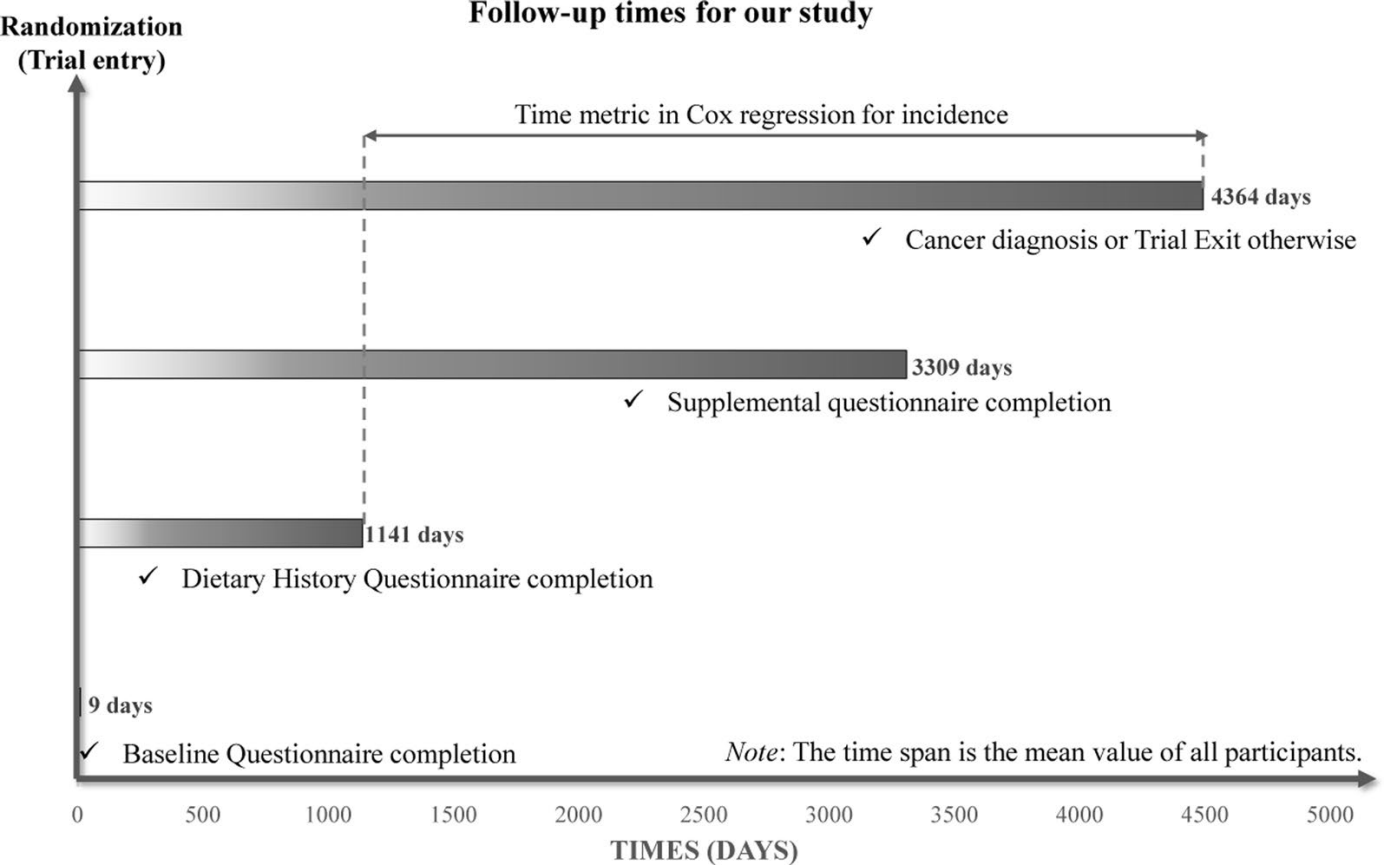
The timeline and follow-up scheme of our study. Notably, in our study, the baseline point was set at the date of diet history questionnaire completion

Cox proportional hazards regression model was used to analyze the hazard ratio (HR) and 95% confidence interval (CI) of PD score, PLL score and CRC incidence, with person-years as the time index. Here, follow-up length was measured from the DHQ completion date to the date of CRC diagnosis, death, loss, or end of follow-up (that is, December 31, 2009), whichever happened first (Fig. 2). To exam whether there was a linear trend between CRC cancer incidence and the quartiles of the two mentioned scores, every individual was assigned a median quartile score within that quartile and subsequently treated as a continuous variable in regression models, using the lowest quartile as the reference group. The P value representing the significance of linear trends. Subsequently, we identified potential confounding variables on the basis of a comprehensive review of the relevant literature and the clinical expertise of the investigators. These variables were included in COX regression models to mitigate their potential impact on the study outcomes [12, 19]. Main model of the potential association between the PD score and risks of CRC was adjusted for demographic features (sex, age, race, and education level), health status (personal history of diabetes, diverticulitis or diverticulosis, colon comorbidity, colorectal polyp, and aspirin use, and a family history of CRC cancer), lifestyle factors (including smoking status, BMI at baseline, and physical activity level), and energy intake value from diet. Given that BMI at baseline, smoking status, and physical activity were used to construct PLL score, these variables were not adjusted in the main model of the association between PLL score and CRC incidence. The same analytical procedures were repeated for the secondary outcome measures, namely the risk of proximal colon cancer and distal colorectal cancer, to investigate the potential association between the incidence of CRC by anatomical subsites and both the PD score and the PLL score. A restricted cubic spline model with three knots at the 10th, 50th, and 90th percentiles was used to illustrate the trends of CRC incidence (including proximal colon cancer and distal colorectal cancer) across the entire range of PD score and PLL score [20]. The reference value was set at the median of the first quartile of PD score and PLL score, which were 35 and 44, respectively. Additionally, the P-nonlinearity was determined by testing the null hypothesis that the regression coefficient of the second spline was equal to zero.

Several prespecified subgroup analyses were performed to test whether the observed associations between PD score and the risk of CRC were modified by age at DHQ completion (> 65 *vs.* ≤ 65 years), sex (male *vs.* female), education levels (high-school graduate or less *vs.* some college or college graduate), family history of CRC cancer (no *vs.* yes/possible), history of diabetes (no *vs.* yes), aspirin use regularly (no *vs.* yes), energy intake from diet (≤ median *vs.* > median, kcal/day), and BMI at baseline (< 30 *vs.* ≥ 30 kg/m^2^). To prevent false subgroup effects, *P*-values for interaction were estimated by comparison of models with and without interaction terms before performing the above-mentioned subgroup analyses. In addition, *P*-values for the trends between quartiles of PD score and CRC incidence were calculated separately for each individual subgroup using the previously described methods. For the PLL score, the same subgroup analyses were performed as described above, whereas the baseline BMI (< 30 *vs.* ≥ 30 kg/m^2^) subgroup was not included.

Additionally, various sensitivity analyses were conducted to assess the robustness of the results. These included: (1) excluding individuals with extreme energy intake (> 4000 kcal/day or < 500 kcal/day); (2) excluding CRC cases that occurred within the first 1 or 2 years of follow-up to investigate the potential impacts of reverse causation; (3) excluding individuals with a history of diabetes, colon comorbidity, colorectal polyps, or a family history of colorectal cancer, as they are at a high risk of developing CRC [12, 21, 22]; (4) adjusting for the Healthy Eating Index-2015 in model 2 to determine whether the observed correlation was influenced by the quality of the diet; and (5) repeating the analysis in the unimputed data cohort to evaluate whether the missing data imputation influenced the results. All statistical analyses were performed using R software version 4.2.2, and a significance level of two-tailed P < 0.05 was used.

## Results

### Participant baseline features

In the whole study population, the mean (standard deviation) was 42 (5) points for PD score, and 51 (7) points for PLL score. The baseline characteristics of the study population according to the quartile of PD score and PLL score are presented in Table 2 and Additional file 1: Table S2, respectively. Participants in the highest quartiles of the PD and PLL score were less likely to be aspirin users, more likely to be females and never smokers, had higher levels of education and physical activity time than those in the lowest quartiles. In addition, compared with those in the lowest quartile of PD and PLL score, those in the highest quartile had lower intake of energy, alcohol, total protein, carbohydrate, and total fat, but higher Healthy Eating Index-2015. Moreover, the proportion of non-White increased with higher quartiles of PD and PLL score. Interestingly, the proportion of individuals with diabetes history increased with increasing quartile of PD score (Table 2), but decreased with higher quartiles of PLL score (Table S2).

**Table 2:** Baseline characteristics of study population according to overall Paleolithic Diet score

Characteristics	Overall	Quartiles of overall paleolithic diet score			
		Quartile 1 (22–37)	Quartile 2 (38–40)	Quartile 3 (41–44)	Quartile 4 (45–63)
Number of participants	74721	17183	14829	20958	21751
Paleolithic diet score	42 ± 5	35 ± 3	39 ± 1	42 ± 1	48 ± 3
Age	65 ± 6	65 ± 6	65 ± 6	65 ± 6	65 ± 6
Female	39251 (53)	5220 (30)	6511 (44)	11719 (56)	15801 (73)
Race					
White (%)	69962 (93.6)	16327 (95.0)	13952 (94.1)	19667 (93.8)	20016 (92.0)
Non-white (%)	4759 (6.4)	856 (5.0)	877 (5.9)	1291 (6.2)	1735 (8.0)
Education level					
High-school graduate or less (%)	20571 (28)	5448 (32)	4390 (30)	5587 (27)	5146 (24)
Some college or college graduate (%)	54150 (73)	11735 (68)	10439 (70)	15371 (73)	16605 (76)
Body mass index at baseline (kg/m^2^)	27 ± 5	27 ± 5	27 ± 5	27 ± 5	27 ± 5
Smoking status					
Never (%)	36566 (49)	7351 (43)	6894 (47)	10538 (50)	11783 (54)
Current (%)	6062 (8.1)	2014 (11.7)	1399 (9.4)	1526 (7.3)	1123 (5.2)
Former (%)	32093 (43)	7818 (46)	6536 (44)	8894 (42)	8845 (41)
Family history of colorectal cancer					
No (%)	65411 (88)	15029 (88)	12951 (87)	18402 (88)	19029 (88)
Yes, or possible (%)	9310 (13)	2154 (13)	1878 (13)	2556 (12)	2722 (13)
History of diverticulitis or diverticulosis (%)	4944 (6.6)	1066 (6.2)	943 (6.4)	1379 (6.6)	1556 (7.2)
History of colon comorbidity (%)	957 (1.3)	195 (1.1)	189 (1.3)	294 (1.4)	279 (1.3)
History of colorectal polyp (%)	4820 (6.5)	1153 (6.7)	1001 (6.8)	1391 (6.6)	1275 (5.9)
History of diabetes (%)	4142 (5.5)	888 (5.2)	794 (5.4)	1201 (5.7)	1259 (5.8)
Aspirin user (%)	34,760 (47)	8298 (48)	6946 (47)	9800 (47)	9716 (45)
Physical activity level (min/week)	130 ± 120	110 ± 120	120 ± 120	130 ± 120	140 ± 130
Energy intake from diet (kcal/day)	1700 ± 720	1900 ± 720	1800 ± 760	1700 ± 740	1600 ± 660
Healthy eating Index-2015	67 ± 10	62 ± 10	65 ± 9	68 ± 9	72 ± 8
Nutrients intakes					
Total calcium (mg/day)	1020 ± 520	960 ± 510	950 ± 520	1010 ± 520	1120 ± 500
Dietary sodium (g/day)	2700 ± 1200	2900 ± 1100	2800 ± 1200	2700 ± 1200	2600 ± 1100
Alcohol (g/day)	10 ± 25	12 ± 29	11 ± 28	9 ± 24	7 ± 18
Total protein (g/day)	67 ± 30	69 ± 29	67 ± 31	67 ± 31	65 ± 29
Carbohydrate (g/day)	220 ± 90	240 ± 88	220 ± 91	220 ± 92	210 ± 85
Total fat (g/day)	63 ± 33	68 ± 33	64 ± 34	62 ± 34	58 ± 31

Descriptive statistics are presented as (mean ± standard deviation) and number (percentage) for continuous and categorical

### Relationship between CRC incidence and paleolithic diet score

During a mean follow-up of 9.2 years (standard deviation: 1.4), a total of 694 CRC cases were reported, including 420 cases of proximal colon cancer, 272 cases of distal CRC cases, and 2 CRC cases with an unknown site. In our study, the overall incidence rate of CRC was 1.01 cases per 1000 person-years. The univariable and multivariable Cox regression analyses results of PD score and the incidence of CRC and its subsites were presented in Table 3. Compared with individuals in the lowest quartile of PD score, those in the highest quartile showed a significantly decreased incidence of CRC, even after adjusting for potential confounders (multivariable model: HR _quartile 4 versus 1_: 0.76; 95% CI 0.61, 0.95; P for trend = 0.009). Similar findings were observed in the analysis of the association between PD score and proximal colon cancer incidence (multivariable model: HR _quartile 4 versus 1_: 0.73; 95% CI 0.55, 0.97; P for trend = 0.02). An inverse association between PD score and distal CRC incidence was also observed, but the result was not statistically significant (multivariable model: HR _quartile 4 versus 1_: 0.82; 95% CI 0.58, 1.16; P for trend = 0.25).

**Table 3:** Hazard Ratios and 95% CIs of Incident Colorectal Cancer in the PLCO Cohort, by Quartiles of Paleolithic Diet score

Model	Paleolithic Diet score, HR (95% CI)				P for trend^a^
	Quartile 1 (lowest)	Quartile 2	Quartile 3	Quartile 4 (highest)	
Colorectal cancer^b^					
Cases	200	148	179	167	NA
Person-years	157688	136768	193197	199074	NA
Incidence rate (95% CI)^c^	1.27 (1.10, 1.46)	1.08 (0.92, 1.27)	0.93 (0.8, 1.07)	0.84 (0.72, 0.98)	NA
Unadjusted	1.00 (reference)	0.85 (0.69, 1.06)	0.73 (0.6, 0.89)	0.66 (0.54, 0.81)	< 0.001
Model 1^d^	1.00 (reference)	0.87 (0.71, 1.08)	0.77 (0.62, 0.94)	0.72 (0.58, 0.90)	0.002
Model 2^e^	1.00 (reference)	0.88 (0.71, 1.09)	0.79 (0.64, 0.97)	0.76 (0.61, 0.95)	0.009
Proximal colon cancer					
Cases	117	94	109	100	NA
Incidence rate (95% CI) ^c^	0.74 (0.62, 0.89)	0.69 (0.56, 0.84)	0.56 (0.47, 0.68)	0.50 (0.41, 0.61)	NA
Unadjusted	1.00 (reference)	0.93 (0.71, 1.21)	0.76 (0.59, 0.99)	0.68 (0.52, 0.88)	0.002
Model 1^d^	1.00 (reference)	0.93 (0.71, 1.22)	0.77 (0.59, 1.00)	0.70 (0.52, 0.92)	0.006
Model 2^e^	1.00 (reference)	0.94 (0.71, 1.23)	0.78 (0.60, 1.02)	0.73 (0.55, 0.97)	0.02
Distal CRC					
Cases	82	54	69	67	NA
Incidence rate (95% CI)^c^	0.52 (0.42, 0.65)	0.39 (0.30, 0.52)	0.36 (0.28, 0.45)	0.34 (0.27, 0.43)	NA
Unadjusted	1.00 (reference)	0.76 (0.54, 1.07)	0.69 (0.50, 0.95)	0.65 (0.47, 0.89)	0.008
Model 1^d^	1.00 (reference)	0.80 (0.57, 1.13)	0.77 (0.55, 1.06)	0.78 (0.55, 1.10)	0.15
Model 2^e^	1.00 (reference)	0.81 (0.58, 1.15)	0.79 (0.57, 1.09)	0.82 (0.58, 1.16)	0.25

a: Trend test was performed using median value of each diet score quintile as a continuous variable

b: Including 420 proximal colon cancer cases, 272 distal CRC (that is, distal colon and rectal cancer) cases, and 2 CRC cases with an unknown site

c: Incidence rate was calculated per 1000 person-years

d: Model 1 was controlled with age (continuous), sex (male, female), race (white, no-white), education levels (high-school graduate or less, some college or college graduate)

e: Model 2 was additionally controlled with family history of colorectal cancer (no, yes or possibly), history of colon comorbidity (no, yes), history of diverticulitis or diverticulosis (no, yes), history of colorectal polyp (no, yes), history of diabetes (no, yes), history of aspirin use (no, yes), total energy intake (continuous), BMI at baseline (continuous), smoking status (never, current, former), and physical activity level (continuous)

*HR* hazard ratio, *CI* confidence interval, *NA* not applicable

**Fig. 3:**
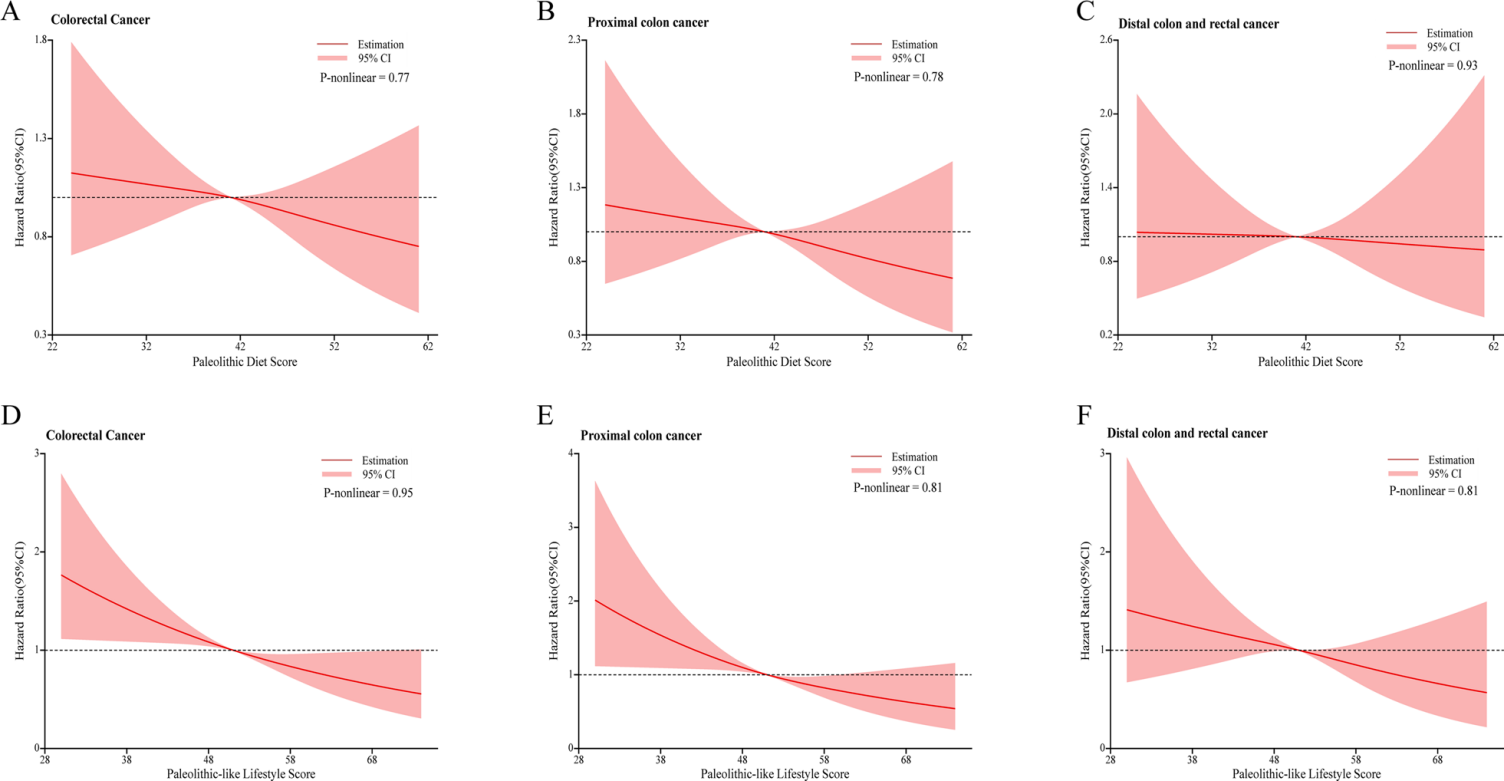
Nonlinear Dose–response analysis on the association of PD score and PLL score with the risk of CRC (A/D: all CRC; B/E: proximal colon cancer; C/F: distal CRC). For PD score, hazard ratio was adjusted for age, sex, race, education levels, family history of colorectal cancer, history of colon comorbidity, history of diverticulitis or diverticulosis, history of colorectal polyp, history of diabetes, history of aspirin use, total energy intake, BMI at baseline, smoking status, and physical activity level. For PLL score, hazard ratio was adjusted for age, sex, race, education levels, family history of colorectal cancer, history of colon comorbidity, history of diverticulitis or diverticulosis, history of colorectal polyp, history of diabetes, history of aspirin use, total energy intake

By using restricted cubic spline regression, the linear inverse dose–response associations between PD score and the incidence of CRC and its subsites were revealed (all P for nonlinearity > 0.05; Fig. 3A, B, C). The subgroup analyses indicated that the inverse association between PD score and CRC risk was consistent across subgroups stratified by age at DHQ completion, sex, education levels, family history of CRC cancer, history of diabetes, aspirin use, energy intake from diet (all P for interaction > 0.05) (Additional file 1: Table S3). However, we observed a stronger inverse association for PD score and CRC risk in individuals with BMI < 30 (HR _quartile 4 versus 1_: 0.68; 95% CI 0.53, 0.87; P for trend = 0.004) compared to those with BMI ≥ 30 (HR _quartile 4 versus 1_: 1.07; 95% CI 0.68, 1.67; P for trend = 0.73) (P for interaction = 0.02). Furthermore, sensitivity analyses showed that the initial correlation between PD score and CRC incidence did not change substantially (Additional file 1**: **Table S4).

### Relationship of paleolithic-like lifestyle score with CRC risk

The association between PLL score and the risk of CRC and its subsites were presented in Table 4**.** We observed a stronger inverse association between CRC and its subsites incidence with PLL score than with PD score in both Model 1 and 2. After adjusting for potential confounders in Model 2, a 36% reduced risk of CRC was discovered in individuals from the highest quartile of PLL score compared with those from the lowest quartile (multivariable model: HR _quartile 4 versus 1_: 0.64; 95% CI 0.51, 0.81; P for trend < 0.001). Likewise, PLL score was inversely associated with the risk of proximal colon cancer (HR _quartile 4 versus 1_: 0.62; 95% CI 0.46, 0.83; P for trend = 0.001) and distal CRC (HR _quartile 4 versus 1_: 0.69; 95% CI 0.48, 0.98; P for trend = 0.03) in the multivariable model (Table 4).

**Table 4:** HR and 95% CIs of Incident Colorectal Cancer in the PLCO Cohort, by Quartiles of Paleolithic-Like Lifestyle score

Model	Paleolithic-Like Lifestyle score, HR (95% CI)				P for trend^a^
	Quartile 1 (lowest)	Quartile 2	Quartile 3	Quartile 4 (highest)	
Colorectal cancer^b^					
Cases	209	165	178	142	NA
Person-years	156836	154630	195776	179486	NA
Incidence rate (95% CI)^c^	1.33 (1.16, 1.53)	1.07 (0.92, 1.24)	0.91 (0.79, 1.05)	0.79 (0.67, 0.93)	NA
Unadjusted	1.00 (reference)	0.80 (0.65, 0.98)	0.68 (0.56, 0.83)	0.59 (0.48, 0.73)	< 0.001
Model 1^d^	1.00 (reference)	0.82 (0.66, 1.00)	0.71 (0.58, 0.87)	0.64 (0.51, 0.80)	< 0.001
Model 2^e^	1.00 (reference)	0.82 (0.67, 1.00)	0.71 (0.58, 0.87)	0.64 (0.51, 0.81)	< 0.001
Proximal colon cancer					
Cases	121	105	110	84	NA
Incidence rate (95% CI)^c^	0.77 (0.65, 0.92)	0.68 (0.56, 0.82)	0.56 (0.47, 0.68)	0.47 (0.38, 0.58)	NA
Unadjusted	1.00 (reference)	0.88 (0.68, 1.14)	0.73 (0.56, 0.94)	0.61 (0.46, 0.80)	< 0.001
Model 1^d^	1.00 (reference)	0.88 (0.67, 1.14)	0.73 (0.56, 0.94)	0.61 (0.46, 0.82)	< 0.001
Model 2^e^	1.00 (reference)	0.88 (0.67, 1.14)	0.73 (0.56, 0.94)	0.62 (0.46, 0.83)	0.001
Distal CRC					
Cases	87	60	68	57	NA
Incidence rate (95% CI)^c^	0.55 (0.45, 0.68)	0.39 (0.30, 0.50)	0.35 (0.27, 0.44)	0.32 (0.25, 0.41)	NA
Unadjusted	1.00 (reference)	0.70 (0.50, 0.97)	0.63 (0.46, 0.86)	0.57 (0.41, 0.80)	0.001
Model 1^d^	1.00 (reference)	0.74 (0.53, 1.02)	0.69 (0.50, 0.95)	0.68 (0.48, 0.97)	0.03
Model 2^e^	1.00 (reference)	0.74 (0.53, 1.03)	0.69 (0.50, 0.96)	0.69 (0.48, 0.98)	0.03

a: Trend test was performed using median value of each diet score quintile as a continuous variable

b: Including 420 proximal colon cancer cases, 272 distal CRC (that is, distal colon and rectal cancer) cases, and 2 CRC cases with an unknown site

c: Incidence rate was calculated per 1000 person-years

d: Model 1 was controlled with age (continuous), sex (male, female), race (white, no-white), education levels (high-school graduate or less, some college or college graduate)

e: Model 2 was additionally controlled with family history of colorectal cancer (no, yes or possibly), history of colon comorbidity (no, yes), history of diverticulitis or diverticulosis (no, yes), history of colorectal polyp (no, yes), history of diabetes (no, yes), history of aspirin use (no, yes), and total energy intake (continuous)

*HR* hazard ratio, *CI* confidence interval, *NA* not applicable

Similar to the previous analysis of PD score, the linear inverse dose–response associations between PLL score and the risk of CRC as well as its subsites was demonstrated (all P for nonlinearity > 0.05; Fig. 3D, E, F). Additional file 1**: **Table S5 presented the result of subgroup analysis for PLL score and CRC incidence. This significant inverse relationship did not alter by pre-defined stratification factors (including age at DHQ completion, sex, education levels, family history of CRC cancer, history of diabetes, aspirin use, energy intake from diet) (all P for interaction > 0.05). The significant inverse relationship between PLL score and CRC incidence persisted in sensitivity analyses (Additional file 1: Table S6).

## Discussion

In this large prospective study, we found that greater adherence to PD or PLL was associated with a lower risk of overall CRC. However, when assessed by anatomical subsites, higher adherence to PD was associated with a lower risk of proximal colon cancer, but not distal CRC. For PLL, the decreased risk in different CRC subsites is similar. In note, using subgroup analysis, we observed a stronger inverse association between PD and CRC risk in individuals with BMI < 30 than those with BMI ≥ 30.

To the best of our knowledge, this study is the first to examine the potential association between PD and PLL and the risk of CRC, as well as its various anatomical subsites, in a large, mixed-gender population. In fact, a previous case–control study has investigated the association between PD and colorectal adenoma, a precursor of CRC [6]. This study recruited 2301 individuals, comprising 564 cases identified through outpatient colonoscopy, 1202 colonoscopy-negative controls, and 535 community controls, and collected dietary information using a Willett FFQ. The study found that the multivariable-adjusted odds ratios (OR) comparing the highest to the lowest quintiles of the PD score were 0.71 (95% CI 0.50–1.02; P for trend = 0.02) for cases versus endoscopy-negative controls, and 0.84 (95% CI 0.56–1.26; P for trend = 0.14) for cases versus community controls. However, no clear differences in the associations were observed according to adenoma anatomical subsites [6]. In another pooled cross-sectional study of a population undergoing elective outpatient colonoscopy (n = 646), Whalen et al. reported that adherence to PD may reduce systemic inflammation and oxidative stress levels in humans [23]. This finding partially supports our conclusion since the progression of CRC is typically associated with inflammation and oxidative stress [24–26]. In addition, epidemiological studies have increasingly confirmed the potential association between adherence to the PD and a reduced risk of other cancers. For instance, Shah et al. observed a 17% lower risk of breast cancer among postmenopausal female with higher adherence to the PD [10].

The following mechanisms can be speculated by which PD lower risk of CRC. First, the PD limits the intake of sugar sweetened beverages, which increased the risk of CRC through pathways of oxidative stress and inflammation [27–29]. Second, previous studies have suggested that the PD may improve insulin sensitivity and prevent diabetes [9, 30, 31], which could potentially reduce the risk of CRC [12, 32]. Extensive evidence supports a direct association of between low insulinemic dietary pattern and reduced risk of total cancer and CRC [33, 34]. Moreover, higher dietary insulin load has been found to be correlated with increased cancer recurrence and poorer survival outcomes among individuals diagnosed with stage III colon cancer [35]. Third, the consumption of red and processed meat, which has been proposed to increase the risk of CRC by producing genotoxic free radicals and inducing lipid peroxidation [36], was restricted in the PD. Forth, restricting sodium and alcohol intake may also have beneficial effects on the pathophysiology of cancer [37–39]. On the other hand, the PD highlights the intake of fruits, vegetables, and nuts, which are abundant sources of dietary fiber and unsaturated fatty acids. These nutrients have been found to exert beneficial effects on modulating detoxification enzymes and the immune system [40–43]. Furthermore, dietary fiber promotes the production of short-chain fatty acids through microbial fermentation, which helps to maintain mucosal integrity and inhibit inflammation and carcinogenesis by affecting immunity and gene expression [44, 45]. Additionally, various prospective studies have suggested that higher intake of calcium or fish may lower the risk of colorectal cancer [46, 47].

Regarding the other three modifiable lifestyle factors of PLL, epidemiological studies have consistently reported direct positive associations between smoking, obesity, and physical inactivity with CRC risk [12]. Physical activity can reduce the risk of CRC by decreasing inflammation, intestinal transit time, insulin-like growth factor levels, hyperinsulinemia, and regulating immune function [48]. The mechanism linking obesity to an increased risk of CRC is that it promotes insulin resistance or hyperinsulinemia, chronic inflammation, oxidative stress, and DNA damage [49]. Smoking primarily plays a role in the development of colorectal cancer by promoting the growth of colorectal cancer cells through nicotine and stimulating angiogenesis in colon cancer [50].

The subgroup analysis revealed an interesting finding: a stronger inverse association between PD score and CRC risk was observed in individuals with BMI < 30 compared to those with BMI ≥ 30. As mentioned earlier, adherence to PD has been shown to increase insulin sensitivity and reduce the risk of diabetes [30, 31], both of which are directly associated with obesity risk [51]. Thus, one of the possible explanations for the phenomenon observed in the subgroup analysis is that individuals who adhere to PD are less likely to be obese since their healthy dietary habits, while obese is directly associated with a high risk of CRC [49]. Another plausible hypothesis is that BMI may mediate the association between adherence to the PD and reduced risk of CRC. Nevertheless, additional investigations are warranted to validate this hypothesis. In addition, we cannot rule out the possibility that the observed interaction between BMI and PD is accidental, although this phenomenon can be explained.

Our study has several notable strengths. First, this study is based on a large-scale prospective cohort of over 150,000 participants recruited from 10 screening centers all over the US. In addition, the follow-up period was calculated from the completion of the DHQ, which ensured an appropriate observation time and for the cohort to obtain enough outcome events. The actual observation time was far greater than the nominal follow-up period of 9 years, as the mean time from randomization to DHQ completion was approximately 3 years. Second, we conducted a comprehensive adjustment for a wide range of potential confounding factors in our analyses. However, we cannot rule out the possibility that there may be additional unmeasured residual confounders that could affect the observed association. Third, our results were supported by a series of sensitivity analyses, which confirmed the robustness of the association between PD and PLL scores and the risk of CRC. Finally, we found an interesting inverse association between PD score and CRC incidence that was more pronounced in participants with a BMI < 30 than in those with a BMI ≥ 30, although the exact reason for this is still unclear.

However, this study has several limitations that should be considered. Firstly, dietary intake was only measured by DHQ at baseline, which did not allow for the assessment of changes in diet over time. Nevertheless, it has been indicated that baseline diet evaluation generally results in weaker associations with disease incidence than cumulative dietary intake [52]. Furthermore, self-reported food frequency questionnaires may not be precise enough due to their extensive contents. Secondly, the modern PD may differ in terms of the nutritional value of the diet of our pre-agricultural ancestors. In addition, as with other dietary pattern analyses, individuals just followed a dietary pattern which was more or less similar to the PD definition, instead of explicitly choosing to adhere to the PD. Finally, the observational design of our study makes it impossible for us to confirm the causal association between PD or PLL and the risk of CRC.

## Conclusions

In conclusion, our study suggests that adherence to the PD or PLL may reduce the risk of CRC. The anatomical subsite analyses showed that PD was inversely associated with the risk of proximal colon cancer, but not distal CRC. However, the reduced risk of different CRC subsites was similar by adhering to PLL. Our results need to be further validated in other populations and settings.

## Supplementary Information

**Additional file 1:  Table S1**. Distribution of covariates with missing data before and after imputation. **Table S2**. Baseline characteristics of study population according to overall Paleolithic-like lifestyle score. **Table S3**. Subgroup analyses on the association between Paleolithic Diet score and colorectal cancer incidence. **Table S4**. Sensitivity analyses on the between Paleolithic Diet score and colorectal cancer incidence. **Table S5**. Subgroup analyses on the association between Paleolithic-like lifestyle score and colorectal cancer incidence. **Table S6**. Sensitivity analyses on the between Paleolithic-like lifestyle score and colorectal cancer incidence.

## Data Availability

The raw data used in this article is not available because of the National Cancer Institute’s data policy. Access to the dataset should contact the National Cancer Institute by mail.

## Abbreviations

BMI: Body mass index
BQ: Baseline questionnaire
CI: Confidence interval
CRC: Colorectal cancer
DHQ: Dietary history questionnaire
HR: Hazard ratio
NCI: National cancer institute
PD: Paleolithic diet
PLCO: Prostate lung colorectal and ovarian
PLL: Paleolithic-like lifestyle
SQX: Supplemental questionnaire
