# Supplementary material for: Adherence to the Paleolithic diet and Paleolithic-like lifestyle reduce the risk of colorectal cancer in the United States: a prospective cohort study
Source: J Transl Med. 2023 Jul 19;21:482. doi: 10.1186/s12967-023-04352-8 (PMC10357623; doi:10.1186/s12967-023-04352-8)
Supplement: Supplementary file 1 — Additional file 1: Table S1. Distribution of covariates with missing data before and after imputation. Table S2. Baseline characteristics of study population according to overall Paleolithic-like lifestyle score. Table S3. Subgroup analyses on the association between Paleolithic Diet score and colorectal cancer incidence. Table S4. Sensitivity analyses on the between Paleolithic Diet score and colorectal cancer incidence. Table S5. Subgroup analyses on the association between Paleolithic-like lifestyle score and colorectal cancer incidence. Table S6. Sensitivity analyses on the between Paleolithic-like lifestyle score and colorectal cancer incidence. [file 12967_2023_4352_MOESM1_ESM.docx]

***Supplementary Material***

**Adherence to the Paleolithic diet and Paleolithic-like lifestyle reduce the risk of colorectal cancer in the United States: A prospective cohort study**

Yi Xiao^1, #^, Yaxu Wang^1, #^, Haitao Gu^1^, Zhiquan Xu^1^, Yunhao Tang^1^, Hongmei He^1^, Linglong Peng^1, *^, Ling Xiang^2, *^

*^1^ Department of Gastrointestinal Surgery, The* *Second Affiliated Hospital of Chongqing Medical University, Chongqing, China.*

*^2^ Department of* *Clinical Nutrition, The Second Affiliated Hospital of Chongqing Medical University, Chongqing, China.*

* Correspondence authors: Linglong Peng (Department of Gastrointestinal Surgery) and Ling Xiang (Department of Clinical Nutrition), The Second Affiliated Hospital of Chongqing Medical University, No.288, Tianwen Avenue, Nan'an District, Chongqing, 400010, China. Fax: +86 023 6288 7521. E-mail addresses: [306359@hospital.cqmu.edu.cn](mailto:306359@hospital.cqmu.edu.cn.com) (Ling Xiang); [penglinglong_cqmu@cqmu.edu.cn](mailto:penglinglong_cqmu@cqmu.edu.cn) (Linglong Peng)

^#^ These authors contributed equally to this work.

***Keywords:*** Paleolithic diet, Paleolithic-like lifestyle, colorectal cancer, epidemiology, cohort study

**List of Supporting Information**

**Supplementary Table 1**. Distribution of covariates with missing data before and after imputation

**Supplementary Table 2**. Baseline characteristics of study population according to overall Paleolithic-like lifestyle score.

**Supplementary Table 3**. Subgroup analyses on the association between Paleolithic Diet score and colorectal cancer incidence

**Supplementary Table 4**. Subgroup analyses on the association between Paleolithic-like lifestyle score and colorectal cancer incidence

**Supplementary Table 5**. Sensitivity analyses on the between Paleolithic Diet score and lung cancer incidence.

**Supplementary Table 6**. Sensitivity analyses on the between Paleolithic-like lifestyle score and lung cancer incidence.

Supplementary Table 1. Distribution of covariates with missing data before and after imputation

| Variable | Before imputation | After imputation | Number (%) with missing data |
| --- | --- | --- | --- |
| Educational level: Some college or college graduate | 54038 (72%) | 54150 (73%) | 112 (0.1%) |
| Aspirin use history: No | 39738 (53%) | 39961 (54%) | 223 (0.3%) |
| Family history of colorectal cancer: No | 64872 (87%) | 65411 (88%) | 539 (0.7%) |
| Diabetes history: No | 70272 (94.4%) | 70579 (94.5%) | 307 (0.4%) |
| Diverticulitis or diverticulosis history: No | 69400 (93.3%) | 69777 (93.4%) | 377 (0.5%) |
| Colon comorbidity history: No | 73248 (98.7%) | 73764 (98.7%) | 516 (0.7%) |
| Colorectal polyp history: No | 69537 (93.5%) | 69901 (93.5%) | 364 (0.5%) |

Values are mean (standard deviation) or counts (percentage) as indicated.

**Supplementary Table 2. Baseline characteristics of study population according to overall Paleolithic-like lifestyle score.**

| **Characteristics** | **Overall** | **Quartiles of overall Paleolithic-like lifestyle score** | | | |
| --- | --- | --- | --- | --- | --- |
|  |  | Quartile 1 (25-46) | Quartile 2 (47-50) | Quartile 3 (51-55) | Quartile 4 (56-76) |
| Number of participants | 74721 | 17181 | 16793 | 21214 | 19533 |
| Paleolithic-like lifestyle score | 51±7 | 43±3 | 49±1 | 53±1 | 60±3 |
| Age (years) | 65±6 | 65±6 | 65±6 | 65±6 | 65±6 |
| Female (%) | 39251 (53) | 5422 (32) | 7484 (45) | 11919 (56) | 14426 (74) |
| Race |  |  |  |  |  |
| White | 69962 (93.6) | 16256 (94.6) | 15845 (94.4) | 19896 (93.8) | 17965 (92.0) |
| Non-white | 4759 (6.4) | 925 (5.4) | 948 (5.6) | 1318 (6.2) | 1568 (8.0) |
| Education level |  |  |  |  |  |
| High-school graduate or less (%) | 20571 (28) | 5765 (34) | 4970 (30) | 5584 (26) | 4252 (22) |
| Some college or college graduate (%) | 54150 (73) | 11416 (66) | 11823 (70) | 15630 (74) | 15281 (78) |
| Body mass index at baseline (kg/m^2^) | 27±5 | 29±5 | 28±5 | 27±5 | 25±4 |
| Smoking status |  |  |  |  |  |
| Never (%) | 36566 (49) | 5095 (30) | 7373 (44) | 11305 (53) | 12793 (66) |
| Current (%) | 6062 (8.1) | 3022 (17.6) | 1510 (9.0) | 1083 (5.1) | 447 (2.3) |
| Former (%) | 32093 (43) | 9064 (53) | 7910 (47) | 8826 (42) | 6293 (32) |
| Family history of colorectal cancer |  |  |  |  |  |
| No (%) | 65411 (88) | 14956 (87) | 14710 (88) | 18590 (88) | 17155 (88) |
| Yes or possible (%) | 9310 (13) | 2225 (13) | 2083 (12) | 2624 (12) | 2378 (12) |
| History of diverticulitis or diverticulosis (%) | 4944 (6.6) | 1128 (6.6) | 1105 (6.6) | 1439 (6.8) | 1272 (6.5) |
| History of colon comorbidity (%) | 957 (1.3) | 208 (1.2) | 210 (1.3) | 283 (1.3) | 256 (1.3) |
| History of colorectal polyp (%) | 4820 (6.5) | 1240 (7.2) | 1132 (6.7) | 1363 (6.4) | 1085 (5.6) |
| History of diabetes (%) | 4142 (5.5) | 1140 (6.6) | 1011 (6.0) | 1175 (5.5) | 816 (4.2) |
| Aspirin user (%) | 34760 (47) | 8520 (50) | 7884 (47) | 9851 (46) | 8505 (44) |
| Physical activity level (min/week) | 130±120 | 75±100 | 110±110 | 130±120 | 180±130 |
| Energy intake from diet (kcal/day) | 1700±720 | 1900±750 | 1800±760 | 1700±730 | 1600±630 |
| Healthy Eating Index-2015 | 67±10 | 61±10 | 65±9 | 68±9 | 72±8 |
| Nutrients intakes |  |  |  |  |  |
| Total calcium (mg/day) | 1020±520 | 940±510 | 950±510 | 1020±520 | 1150±500 |
| Dietary sodium (g/day) | 2700±1200 | 2900±1200 | 2800±1200 | 2700±1200 | 2600±1100 |
| Alcohol (g/day) | 10±25 | 13±32 | 11±26 | 9±23 | 6±15 |
| Total protein (g/day) | 67±30 | 69±30 | 68±31 | 67±31 | 64±28 |
| Carbohydrate (g/day) | 220±90 | 230±90 | 220±91 | 220±91 | 210±85 |
| Total fat (g/day) | 63±33 | 69±34 | 65±35 | 61±33 | 56±29 |

Descriptive statistics are presented as mean ± standard deviation and number (percentage) for continuous and categorical.

|  |  |  | **Paleolithic Diet score, HR (95% CI)** | | | |  |  |
| --- | --- | --- | --- | --- | --- | --- | --- | --- |
| **Subgroup variable** | **Cases** | **Person-years** | **Quartile 1** | **Quartile 2** | **Quartile 3** | **Quartile 4** | ***P* _trend_ ^a^** | ***P* _interaction_ ^b^** |
| Age (years) |  |  |  |  |  |  |  | 0.83 |
| ≤65 | 269 | 372923 | 1.00 (reference) | 0.94 (0.66, 1.33) | 0.80 (0.57, 1.12) | 0.82 (0.58, 1.16) | 0.21 |  |
| >65 | 425 | 313804 | 1.00 (reference) | 0.84 (0.64, 1.11) | 0.77 (0.60, 1.00) | 0.72 (0.55, 0.96) | 0.02 |  |
| Sex |  |  |  |  |  |  |  | 0.24 |
| Male | 379 | 327129 | 1.00 (reference) | 1.02 (0.79, 1.33) | 0.88 (0.67, 1.15) | 0.84 (0.61, 1.16) | 0.20 |  |
| Female | 315 | 359598 | 1.00 (reference) | 0.65 (0.45, 0.93) | 0.62 (0.45, 0.86) | 0.61 (0.45, 0.83) | 0.02 |  |
| Education levels |  |  |  |  |  |  |  | 0.33 |
| High-school graduate or less | 214 | 188641 | 1.00 (reference) | 0.74 (0.51, 1.09) | 0.68 (0.47, 0.99) | 0.83 (0.57, 1.21) | 0.27 |  |
| Some college or college graduate | 480 | 498086 | 1.00 (reference) | 0.96 (0.74, 1.24) | 0.84 (0.65, 1.08) | 0.74 (0.56, 0.96) | 0.02 |  |
| Family history of colorectal cancer |  |  |  |  |  |  |  | 0.39 |
| No | 597 | 601063 | 1.00 (reference) | 0.94 (0.75, 1.18) | 0.81 (0.65, 1.01) | 0.75 (0.59, 0.95) | 0.01 |  |
| Yes, or possible | 97 | 85664 | 1.00 (reference) | 0.60 (0.33, 1.10) | 0.64 (0.37, 1.11) | 0.80 (0.46, 1.39) | 0.44 |  |
| Diabetes history |  |  |  |  |  |  |  | 0.84 |
| No | 633 | 649361 | 1.00 (reference) | 0.87 (0.69, 1.08) | 0.79 (0.64, 0.98) | 0.75 (0.59, 0.94) | 0.009 |  |
| Yes | 61 | 37366 | 1.00 (reference) | 1.11 (0.53, 2.33) | 0.79 (0.38, 1.66) | 0.89 (0.43, 1.84) | 0.63 |  |
| Aspirin use regularly |  |  |  |  |  |  |  | 0.41 |
| No | 376 | 368893 | 1.00 (reference) | 0.82 (0.61, 1.11) | 0.84 (0.64, 1.10) | 0.69 (0.51, 0.93) | 0.02 |  |
| Yes | 318 | 317834 | 1.00 (reference) | 0.95 (0.70, 1.30) | 0.72 (0.53, 0.98) | 0.85 (0.62, 1.17) | 0.18 |  |
| Energy intake from diet (kcal/day) |  |  |  |  |  |  |  | 0.82 |
| ≤median ^c^ | 343 | 342965 | 1.00 (reference) | 0.79 (0.57, 1.08) | 0.69 (0.51, 0.94) | 0.68 (0.50, 0.93) | 0.02 |  |
| >median | 351 | 343762 | 1.00 (reference) | 0.96 (0.72, 1.28) | 0.87 (0.65, 1.15) | 0.82 (0.60, 1.12) | 0.17 |  |
| BMI at baseline (kg/m^2^) |  |  |  |  |  |  |  | 0.02 |
| ＜30 | 532 | 535358 | 1.00 (reference) | 0.77 (0.60, 0.99) | 0.80 (0.64, 1.01) | 0.68 (0.53, 0.87) | 0.004 |  |
| ≥30 | 162 | 151370 | 1.00 (reference) | 1.32 (0.87, 2.02) | 0.70 (0.44, 1.12) | 1.07 (0.68, 1.67) | 0.73 |  |

**Supplementary Table 3. Subgroup analyses on the association between Paleolithic Diet score and colorectal cancer incidence**

Abbreviations: HR, hazard ratio; CI, confidence interval;

**a:** Trend test was performed using median value of each diet score quintile as a continuous variable.

**b**: P value for interaction was estimated using the likelihood ratio test comparing the model with and without the interaction term of the paleolithic diet score and the respective stratification variable.

**c**: The median of dietary energy intake in this study is 1613 kcal/day.

Hazard ratios were adjusted for age (continuous), sex (male, female), race (white, no-white), education levels (high-school graduate or less, some college or college graduate), family history of colorectal cancer (no, yes or possibly), history of colon comorbidity (no, yes), history of diverticulitis or diverticulosis (no, yes), history of colorectal polyp (no, yes), history of diabetes (no, yes), history of aspirin use (no, yes), total energy intake (continuous), BMI at baseline (continuous), smoking status (never, current, former), and physical activity level (continuous).

**Supplementary Table 4. Sensitivity analyses on the between Paleolithic Diet score and lung cancer incidence.**

| **Categories** | **Participants** | **Cases** | **HR Quartile 4 vs. Quartile 1 (95% CI) ^a^** | ***P* _trend_** |
| --- | --- | --- | --- | --- |
| Primary analysis | 74721 | 694 | 0.76 (0.61, 0.95) | 0.009 |
| Excluded participants with extreme energy intake ^b^ | 73725 | 679 | 0.75 (0.60, 0.94) | 0.007 |
| Excluded cases observed within the first 1 years of follow-up | 74664 | 637 | 0.77 (0.61, 0.96) | 0.01 |
| Excluded cases observed within the first 2 years of follow-up | 74601 | 574 | 0.78 (0.62, 1.00) | 0.03 |
| Excluded participants with diabetes | 70579 | 633 | 0.75 (0.59, 0.94) | 0.009 |
| Excluded participants with colon comorbidity | 73764 | 684 | 0.77 (0.62, 0.96) | 0.01 |
| Excluded participants with colorectal polyp | 69901 | 641 | 0.80 (0.63, 1.00) | 0.04 |
| Excluded participants with family history of colorectal cancer | 65411 | 597 | 0.75 (0.59, 0.95) | 0.01 |
| Further adjusted for Healthy Eating Index-2015 | 74721 | 694 | 0.77 (0.61, 0.97) | 0.02 |
| Repeated analysis in unimputed data cohort | 74721 | 694 | 0.75 (0.60, 0.94) | 0.007 |

Abbreviations: HR, hazard ratio; CI, confidence interval;

**a**: Hazard ratios were adjusted for age (continuous), sex (male, female), race (white, no-white), education levels (high-school graduate or less, some college or college graduate), family history of colorectal cancer (no, yes or possibly), history of colon comorbidity (no, yes), history of diverticulitis or diverticulosis (no, yes), history of colorectal polyp (no, yes), history of diabetes (no, yes), history of aspirin use (no, yes), total energy intake (continuous), BMI at baseline (continuous), smoking status (never, current, former), and physical activity level (continuous).

**b**: The extreme energy intake was defined as >4000 kcal/day or <500 kcal/day.

|  |  |  | **Paleolithic-like lifestyle score, HR (95% CI)** | | | |  |  |
| --- | --- | --- | --- | --- | --- | --- | --- | --- |
| **Subgroup variable** | Cases | Person-years | Quartile 1 | Quartile 2 | Quartile 3 | Quartile 4 | ***P* _trend_** ^a^ | ***P* _interaction_** ^b^ |
| Age (years) |  |  |  |  |  |  |  | 0.67 |
| ≤65 | 269 | 372923 | 1.00 (reference) | 0.71 (0.51, 0.99) | 0.67 (0.48, 0.93) | 0.64 (0.45, 0.91) | 0.008 |  |
| >65 | 425 | 313804 | 1.00 (reference) | 0.89 (0.69, 1.16) | 0.74 (0.57, 0.96) | 0.65 (0.48, 0.87) | 0.001 |  |
| Sex |  |  |  |  |  |  |  | 0.06 |
| Male | 379 | 327129 | 1.00 (reference) | 0.98 (0.76, 1.25) | 0.72 (0.55, 0.95) | 0.79 (0.57, 1.09) | 0.03 |  |
| Female | 315 | 359598 | 1.00 (reference) | 0.57 (0.40, 0.81) | 0.62 (0.46, 0.85) | 0.50 (0.37, 0.69) | <0.001 |  |
| Education levels |  |  |  |  |  |  |  | 0.14 |
| High-school graduate or less | 214 | 188641 | 1.00 (reference) | 0.67 (0.46, 0.97) | 0.75 (0.52, 1.07) | 0.82 (0.55, 1.22) | 0.20 |  |
| Some college or college graduate | 480 | 498086 | 1.00 (reference) | 0.89 (0.70, 1.14) | 0.70 (0.54, 0.89) | 0.59 (0.45, 0.78) | <0.001 |  |
| Family history of colorectal cancer |  |  |  |  |  |  |  | 0.71 |
| No | 597 | 601063 | 1.00 (reference) | 0.79 (0.63, 0.98) | 0.71 (0.57, 0.88) | 0.61 (0.48, 0.78) | <0.001 |  |
| Yes, or possible | 97 | 85664 | 1.00 (reference) | 1.01 (0.60, 1.72) | 0.68 (0.38, 1.20) | 0.84 (0.46, 1.51) | 0.35 |  |
| Diabetes history |  |  |  |  |  |  |  | 0.84 |
| No | 633 | 649361 | 1.00 (reference) | 0.79 (0.63, 0.98) | 0.73 (0.59, 0.90) | 0.64 (0.51, 0.81) | <0.001 |  |
| Yes | 61 | 37366 | 1.00 (reference) | 1.11 (0.59, 2.09) | 0.51 (0.24, 1.08) | 0.72 (0.33, 1.54) | 0.15 |  |
| Aspirin use regularly |  |  |  |  |  |  |  | 0.45 |
| No | 376 | 368893 | 1.00 (reference) | 0.71 (0.53, 0.95) | 0.71 (0.54, 0.94) | 0.60 (0.44, 0.81) | 0.001 |  |
| Yes | 318 | 317834 | 1.00 (reference) | 0.95 (0.71, 1.27) | 0.70 (0.51, 0.95)) | 0.70 (0.50, 0.98) | 0.01 |  |
| Energy intake from diet (kcal/day) |  |  |  |  |  |  |  | 0.48 |
| ≤median ^c^ | 343 | 342965 | 1.00 (reference) | 0.82 (0.61, 1.11) | 0.60 (0.44, 0.81) | 0.60 (0.44, 0.82) | 0.001 |  |
| >median | 351 | 343762 | 1.00 (reference) | 0.79 (0.60, 1.06) | 0.82 (0.62, 1.07) | 0.68 (0.49, 0.94) | 0.02 |  |

**Supplementary Table 5. Subgroup analyses on the association between Paleolithic-like lifestyle score and colorectal cancer incidence**

Abbreviations: HR, hazard ratio; CI, confidence interval;

**a:** Trend test was performed using median value of each diet score quintile as a continuous variable.

**b**: P value for interaction was estimated using the likelihood ratio test comparing the model with and without the interaction term of the paleolithic diet score and the respective stratification variable.

**c**: The median of dietary energy intake in this study is 1613 kcal/day.

Hazard ratios were adjusted for age (continuous), sex (male, female), race (white, no-white), education levels (high-school graduate or less, some college or college graduate), family history of colorectal cancer (no, yes or possibly), history of colon comorbidity (no, yes), history of diverticulitis or diverticulosis (no, yes), history of colorectal polyp (no, yes), history of diabetes (no, yes), history of aspirin use (no, yes), and total energy intake (continuous).

| **Categories** | **Participants** | **Cases** | **HR Quartile 4 vs. Quartile 1 (95% CI) ^a^** | ***P* _trend_** |
| --- | --- | --- | --- | --- |
| Primary analysis | 74721 | 694 | 0.64 (0.51, 0.81) | <0.001 |
| Excluded participants with extreme energy intake ^b^ | 73725 | 679 | 0.65 (0.52, 0.82) | <0.001 |
| Excluded cases observed within the first 1 years of follow-up | 74664 | 637 | 0.65 (0.51, 0.82) | <0.001 |
| Excluded cases observed within the first 2 years of follow-up | 74601 | 574 | 0.65 (0.50, 0.83) | <0.001 |
| Excluded participants with diabetes | 70579 | 633 | 0.64 (0.51, 0.81) | <0.001 |
| Excluded participants with colon comorbidity | 73764 | 684 | 0.66 (0.52, 0.82) | <0.001 |
| Excluded participants with colorectal polyp | 69901 | 641 | 0.66 (0.52, 0.83) | <0.001 |
| Excluded participants with family history of colorectal cancer | 65411 | 597 | 0.61 (0.48, 0.78) | <0.001 |
| Further adjusted for Healthy Eating Index-2015 | 74721 | 694 | 0.67 (0.52, 0.85) | 0.001 |
| Repeated analysis in unimputed data cohort | 74721 | 694 | 0.63 (0.50, 0.80) | <0.001 |

**Supplementary Table 6. Sensitivity analyses on the between Paleolithic-like lifestyle score and lung cancer incidence.**

Abbreviations: HR, hazard ratio; CI, confidence interval;

**a**: Hazard ratios were adjusted for age (continuous), sex (male, female), race (white, no-white), education levels (high-school graduate or less, some college or college graduate), family history of colorectal cancer (no, yes or possibly), history of colon comorbidity (no, yes), history of diverticulitis or diverticulosis (no, yes), history of colorectal polyp (no, yes), history of diabetes (no, yes), history of aspirin use (no, yes), and total energy intake (continuous).

**b**: The extreme energy intake was defined as >4000 kcal/day or <500 kcal/day.
